# Vertebrate Vitellogenin Gene Duplication in Relation to the “3R Hypothesis”: Correlation to the Pelagic Egg and the Oceanic Radiation of Teleosts

**DOI:** 10.1371/journal.pone.0000169

**Published:** 2007-01-24

**Authors:** Roderick Nigel Finn, Børge A. Kristoffersen

**Affiliations:** Department of Biology, University of Bergen, Bergen, Norway; University of Würzburg, Germany

## Abstract

The spiny ray-finned teleost fishes (Acanthomorpha) are the most successful group of vertebrates in terms of species diversity. Their meteoric radiation and speciation in the oceans during the late Cretaceous and Eocene epoch is unprecedented in vertebrate history, occurring in one third of the time for similar diversity to appear in the birds and mammals. The success of marine teleosts is even more remarkable considering their long freshwater ancestry, since it implies solving major physiological challenges when freely broadcasting their eggs in the hyper-osmotic conditions of seawater. Most extant marine teleosts spawn highly hydrated pelagic eggs, due to differential proteolysis of vitellogenin (Vtg)-derived yolk proteins. The maturational degradation of Vtg involves depolymerization of mainly the lipovitellin heavy chain (LvH) of one form of Vtg to generate a large pool of free amino acids (FAA 150–200 m*M*). This organic osmolyte pool drives hydration of the ooctye while still protected within the maternal ovary. In the present contribution, we have used Bayesian analysis to examine the evolution of vertebrate Vtg genes in relation to the “3R hypothesis” of whole genome duplication (WGD) and the functional end points of LvH degradation during oocyte maturation. We find that teleost Vtgs have experienced a post-R3 lineage-specific gene duplication to form paralogous clusters that correlate to the pelagic and benthic character of the eggs. Neo-functionalization allowed one paralogue to be proteolyzed to FAA driving hydration of the maturing oocytes, which pre-adapts them to the marine environment and causes them to float. The timing of these events matches the appearance of the Acanthomorpha in the fossil record. We discuss the significance of these adaptations in relation to ancestral physiological features, and propose that the neo-functionalization of duplicated Vtg genes was a key event in the evolution and success of the teleosts in the oceanic environment.

## Introduction

### Ancestral Gene Landscapes

Gene duplication has long been recognized as a powerful mechanism in evolution [1]–[3]. As noted by Haldane [1], and Ohno [2] the products of such events may acquire new functions (neo-functionalization), survive due to dosage effects (sub-functionalization), become dysfunctional due to mutational insertions, deletions and/or substitutions (pseudogenes), or be lost, without obligate deleterious effects to the host. Whole genome duplications (WGD) have been argued to be the bases of major events in the evolution of vertebrates [2], [4]–[15]. However, the massive gene loss that may occur in the aftermath of WGD, can mask the ancestral ploidy of an organism [2], [16]–[18]. Similarly the propensity for certain lineages to acquire transposable elements; to independently duplicate genes through cis- or trans- events; to retain or lose extra chromosomes (aneuploidy) or a complete set of chromosomes (tetraploidy, haploidy, diploidization) during meiosis or early germline mitosis [17] with subsequent deletion or translocation of genes or chromosomes, further complicates the deciphering of ancestral gene landscapes [4], [16], [19], [20]. Indeed, while teleosts are considered to have doubled their genes in relation to humans (see below), chromosomal loss, or diploidization [16], [17] seems to have been rampant in this group, since similar numbers exist between teleosts (∼20–25) and rodents and primates (19–22 autosomes). As a result, the number of WGD, and whether such events have occurred at all, has remained controversial [9], [12], [15], [18], [21]–[25].

Recent comparative genomic analyses of paralogous chromosomal regions (paralogons) between humans and other vertebrate and invertebrate model organisms have provided convincing evidence that two rounds of WGD occurred during early chordate evolution [8], [12], [20], [26]. Similar analyses between humans and the gene maps of actinopterygian fishes revealed a double conserved synteny between the chromosomes of the teleosts compared to those of a human [11], [27]–[30], and thus demonstrated that a fish-specific WGD first postulated by Ohno [2] and later proposed by Amores *et al*. [5] had indeed occurred. Most recently, Crow *et al*. [31] provided strong evidence that the fish-specific WGD took place after the separation of the crown group of teleosts from bowfin, a neopterygian fish. Hence three rounds (R1, R2, R3) of WGD have occurred during the evolution of teleost fishes from early chordate roots, and traces of these ancient events should be detectable in the many thousands of extant species [13], [32]–[34]. Any phylogenetic reconstruction of genes should thus reconcile the chronology of these events with the proposed tree model and the fossil record.

### A Brief History of Teleosts

The origin of the Actinopterygii dates back to paleonisciform fishes during the late Silurian (410 mya) some 40 million years after separating from the Sarcopterygii [35]–[39]. One of the most primitive actinopterygian fishes was *Cheirolepis*, a freshwater fish that did not enter seawater [37], [40]. While some descendents, such as *Mimia* and *Bigeria* did apparently enter seawater, their lines became extinct long ago and thus did not contribute to the teleost lineage. Only those groups that remained in freshwater exist today. These are the Chondrostei including the Polypteriformes (bichirs) and Acipenseriformes (sturgeons and paddlefishes), and the neopterygian Semionotiformes (gars) and Amiiformes (bowfin). Despite anadromic behaviour in sturgeons, all of these fishes are obligate freshwater spawners, as indeed are the more ancestral Hyperoartia (lampreys). The monophyletic primitive teleosts [41], [42] did not appear in the fossil record until 235 mya [37], but are suggested to have arisen 250–290 mya [32], [39], [43]–[46]. Despite some teleost fossils being recorded from marine deposits, it is not known whether this is the result of anadromous behaviour, or represents true marine species spawning in the ocean. The oldest modern teleosts, however, are the Osteoglossomorpha (bony tongues), all of which, both extinct and extant, were and are freshwater species [44], [47]. The first extant modern marine teleosts appeared as Elopomorpha (e.g. eels and tarpons) during the Jurassic while subsequent Clupeomorpha (e.g. herrings and shads) had both freshwater and anadromous forms. The most successful group of freshwater teleosts, the Ostariophysi (carps and catfishes), were the next to appear (∼245 mya, [39]), and today represent more than 75% of all freshwater fishes of the world with ∼8000 species (30% of all teleosts) [47]–[49]. However, they only achieved their great diversity in freshwater long after the appearance of the Acanthomorpha [37]. It was not until the appearance of Acanthomorpha ∼55–125 mya that the unprecedented radiation and speciation of the teleosts occurred. Extant Acanthomorpha comprise ∼16,000 species, equivalent to 86% of non-ostariophysan teleosts [47], (and see supplementary material Fig. S1). According to Maissey [37]

*“Charts showing the range of fishes through geological time suddenly sprout hundreds of new families. Many of the fossils come from the marine strata of Monte Bolca in Northern Italy. These and other fossils from around the world leave no doubt that modern family-level acanthomorph diversity is rooted in the early Eocene (about 55 million yeas ago), and that this teleost explosion was the most dramatic evolutionary radiation ever seen in vertebrate history, eclipsing the evolution of mammals and birds in numbers of families and species.”*

The basis of this teleost explosion has never been satisfactorily explained. Although the long freshwater ancestry of the teleosts is documented in the fossil record, it can also be inferred from their physiology. All teleosts are hypo-osmotic to seawater, and hyper-osmotic to freshwater. The hypo-osmotic condition of marine teleosts as a group is unique among animal taxa, and is generally assumed to reflect their freshwater past [50], [51]. A related argument for a freshwater origin concerns the presence of a glomerular kidney and a distal tubule segment, the main functions of which are to produce copious volumes of hypo-osmotic urine to counteract the osmotic influx of water in the freshwater teleost [52], [53]. Glomerular kidneys are also present in the more ancient Chondrichthyes, with glomerular filtration rates (GFR: 1–4 mL • kg^−1^ • h^−1^) equivalent to the GFRs of freshwater teleosts (2–10 mL • kg^−1^ • h^−1^) [53]–[55]. Indeed the presence of glomerular kidneys led Homer Smith [52] to suggest that the Chondrichthyes, and even the extinct placoderms, had a freshwater origin. He [52] argued that the glomerulus, the sole function of which is to filter extracellular fluids, could only have arisen in the presence of excess water – i.e. freshwater. In contrast, the marine teleosts have considerably reduced glomeruli, and fractional GFRs (0.1–0.5 mL • kg^−1^ • h^−1^) compared to their freshwater relatives and the Chondrichthyes. Some marine teleosts are even aglomerular (a unique condition among vertebrata), relying entirely on secretion rather than filtration for ion regulation [51]–[53]. Reduction or loss of glomeruli may be considered an adaptation facilitating the conservation of water in the functionally arid marine environment.

### Spawning in the Sea – the Water Problem

The long freshwater ancestry of teleosts implies that also their eggs, which are freely broadcast into the environment by their oviparous parents, had become adapted to the freshwater conditions in the rivers and lakes. When, however, the teleosts eventually started to spawn in the sea, the eggs met new, opposite osmotic problems. The yolk osmolarity is similar to the parental body fluids, ∼350 m*Osm* [56], [57], and thus hypo-osmotic to seawater. Hence, instead of an osmotic influx, the problem in seawater is a continuous osmotic water efflux. Thus, since the osmoregulatory systems (mitochondrial rich cells, intestine, kidneys, and gills) are not yet developed, the egg must be endowed with a water reservoir before it can be spawned in the hyper-osmotic seawater. A water reservoir in the egg is a prerequisite and a necessary pre-adaptation before the teleosts could complete their life cycles in the oceans and truly establish themselves as marine organisms. Without this egg feature, the adult fish had to remain anadromous and return to their freshwater habitats to spawn their eggs. Indeed anadromy is typical of the more primitive fishes including lampreys, sturgeons, shads and salmonids.

Already Fulton [58], [59] and Milroy [60] noted the remarkable volume increase during oocyte maturation in the eggs of marine teleosts, and intuitively proposed that an osmotic mechanism caused a watery fluid to be secreted into the oocytes. This unique oocyte hydration in marine teleosts has been confirmed for a wide range of species with the degree of hydration being much greater in species that spawn pelagic eggs (pelagophils) compared to those that spawn benthic eggs (benthophils). In benthophils the mechanism mostly involves the differential movement of inorganic ions, and a high concentration of the amino acid analogue taurine, while in pelagophils, free amino acids (FAA) due to the maturational proteolysis of vitellogenin (Vtg)-derived yolk proteins, are the main osmolytes driving the hydration [56], [57], [61]–[74]. This growing body of literature shows that up to three forms of Vtg are expressed and differentially processed in pelagophil and benthophil teleosts.

### Vitellogenins and the Organic Osmolyte Pool of Pelagic Eggs

In teleosts Vtg genes are linearly organized as large monomeric structures with multiple sub-domains consisting of a lipovitellin heavy chain (LvH), phosvitin (Pv), lipovitellin light chain (LvL), and a von Willebrand factor type D domain (vWFD) that is split into a beta-component (ß′) and a C-terminal coding region (CT). Once assembled and secreted by the liver, Vtgs are taken up via clathrin-mediated endocytosis by the growing oocytes and cleaved by cathepsin D (CatD) in the early endosomes to form the primary yolk proteins [75]–[78]. The CatD processing represents the primary cleavage event in the degradation of yolk proteins. Recent studies have shown that developmentally regulated V-class ATPases (proton pumps) acidify the yolk platelets during oocyte maturation [66], [79], [80]. This disassembles the crystalline, or in some species non-crystalline yolk and activates other cathepsins (CatL or CatB, dependent upon species) that hydrolytically attack the yolk proteins [75], [76] [66], [77], [80], [81]. This latter processing is known as the secondary cleavage event and is unique to marine teleosts. In some freshwater species, such as zebrafish, electrophoretic band-shifts of the LvH occurs during oocyte maturation due to nicking [82], but no proteolysis, and no buildup of FAA occurs (Finn, unpublished data), which would be osmotically disadvantageous to the freshwater embryo. During oocyte maturation in marine benthophils the yolk proteins are either not processed, or are partially cleaved and hydrolyzed with the release of a small pool of FAA [69], [73], while in marine pelagophils, the yolk proteins are not only cleaved but undergo extensive differential proteolysis of particularly one of the LvH domains resulting in the buildup of the large pool of FAA. The transient increase in osmolarity during oocyte maturation [57] causes water influx via specialized aquaporins that are temporally inserted in the plasma membrane during this period [83], [84]. This hydration provides the early embryo of marine teleosts with a vital water reservoir before a drinking mechanism is developed [57].

Fulton [59] also noted that the greater hydration of the pelagic eggs compared to the benthic eggs caused them to float and hence acquire their pelagic nature. More recently, in a comprehensive review of the early life history stages of fishes and their characters, Kendall *et al*. [85] revealed that most extant marine fishes, regardless of systematic affinities, demersal or pelagic habits, coastal or oceanic distribution, tropical or boreal ranges, spawn pelagic eggs (see also supplementary material, Fig. S1). These findings suggest that the rise of the pelagic egg was an important event in the evolution of the teleosts.

### Vitellogenin Genealogy

Since no evidence of the ancestral spawning habits of teleost fishes has been recorded in the fossil record, we can only infer it. The best means of achieving this is through Bayesian phylogenetic inference [86]–[88] for genes involved in the reproductive physiology of the parent and the survival of the embryo. The hill-climbing, and proportional hill-hopping Markov Chain Monte Carlo algorithm of Bayesian methods is superior to traditional methods of phylogentic inference [87]. Since Vtg-derived yolk proteins are the major components of the egg that sustain the embryo during early development, and are implicated in the pre-adaptation to the marine environment, we analyzed all available vertebrate Vtg genes (sequenced and genomic) and the related homologue apolipoprotein B (apoB) in relation to WGD. Previous studies have shown that apoB, which is related to Vtgs [89]–[91], is also incorporated in the yolk of birds [92], and may represent a neo-functional product of gene duplication. We therefore included the first ∼1000 amino acids of apoB proteins, which represent the large lipid transfer module (LLTM) that is homologous the LvH of Vtgs. Other studies have claimed or continue to cite that the phosvitinless class of Vtg, which lacks the polyserine domain, is more closely related to insect Vtgs [93]–[95]. We therefore included cnidarian and molluscan Vtgs, which predate the arthropods, as outgroups in order to reconcile the topology of the tree with the fossil record, currently accepted phylogenies, and the “3R hypothesis”.

The notion that R3, which is estimated to have occurred prior to the appearance of the crown group of teleosts >290 mya [11], [15], [32], , is the primary cause of the diversity of teleosts seems improbable due to the ∼200 million year gap between the WGD and the rapid diversification. Based upon the present analyses, we propose that the radiation and speciation of the acanthomorph teleosts is rooted in the adaptation of their eggs to the marine environment. We argue that the origin of the LLTM, which predates the bilateria [96], is the molecular harbinger of key adaptations that facilitated the dramatic radiation, while retention of 3R gene products latently contributed to speciation of the Acanthomorpha during the late Cretaceous and Eocene epochs.

## Materials and Methods

### Sequences

Based on our recent sequencing of full length Vtg genes from haddock [67] and Atlantic halibut (Finn, unpublished data), related members of this family of low density lipoproteins were identified using the NCBI BLAST interfaces (www.ncbi.nlm.nih.gov/BLAST). Homologies were confirmed using the blast 2 sequence tool [97]. Using this approach we identified 38 full length vertebrate Vtg homologues, and 56 partial sequences of which only 10 salmonid sequences were included due to their sufficient length and position at the N-terminal domain. We further accessed all available Vtg constructs for vertebrates from the Ensembl genome database (www.ensembl.org). This resulted in the inclusion of 8 more constructs from chicken, zebrafish, 3-spined stickleback, medaka and torafugu. In addition we included the first ∼1000 amino acids of apoB proteins from 5 model organisms. In total ∼83, 000 aa were aligned in 73 sequences.

Since there is some debate in the literature as to the antiquity of the phosvitinless Vtg genes [93], [95], [98], two invertebrates (galaxy coral and Pacific oyster) were used as outgroups. All sequences studied are summarized in Table S1.

In order to corroborate these analyses and confirm the number of gene forms within teleosts, we retrieved all available Vtg genes from currently sequenced teleost genomes (www.ensembl.org). This resulted in separate analyses of 2 genes from torafugu, 11 genes from 3-spined stickleback, 6 genes from medaka, and more than 16 genes from zebrafish. Due to recent updates of the zebrafish genome, we included both the new fragments from Ensembl release 41 (October 2006), and full genes from release 38 (April, 2006) to match the published data of Wang *et al*. [98].

### Multiple Sequence Alignments

Multiple amino acid (aa) alignments of the Vtg homologues were achieved using several programs. Initial alignments were performed on the full length Vtg protein data set with default settings using t-coffee, Clustal W, Muscle and Probcons [99]–[102]. Extensive modelling was performed using Blossum and PAM matrices and by varying gap-open and gap-extension penalties. This strategy allowed us to make a high quality alignment for the first ∼1075 aa, and the last ∼500 aa. The remaining polyserine segment was separately aligned using t-coffee and re-inserted into the full alignment. In addition, we further modelled the alignment in Clustal X profile mode using the lamprey structure mask for Vtg [103], [104]. This latter approach allowed us to minimize gaps in regions associated with secondary structures. Based on these outputs we manually adjusted the sequences to give a final full length multiple alignment. A second data set included the 10 partial salmonid sequences that were added to the full data set using Clustal X in profile mode. All data sets were then converted to codon alignments using the University of Bergen computational biology unit alignment to coding tool (www.bioinfo.no).

To determine the most likely tree topology, the full aa and codon alignments were analyzed using phylogenetic programs, and then re-examined after removal of the signal peptides, and polyserine domain which showed the least consensus. The alignments were further examined after removal of regions that contained gaps in more than 70% of the taxa, and again after removal the C-terminal vWFD domain that only occurs in most VtgA type homologues. Two separate sets of the first 270 aa and 810 nucleic acids, which included the partial salmonid sequences, with and without gaps were also analysed. A summary of the domains analysed is shown below the multiple sequence alignment (see supplementary information, Fig. S2).

### Phylogenetic Analyses

Bayesian analyses (MrBayes 3.1.2; [105]) was used for each of the aligned aa and codon data sets. The following settings were used for codon alignments: nucmodel = 4by4, nst = 2, rates = gamma; and aa alignments: aamodel = mixed, with 1,000,000 generations, sampled every 100 generations using 4 chains and a burnin of 3,500. For each run, a Majority rule consensus tree together with posterior probabilities from the last 6,500 trees, representing 650,000 generations, was rendered with ATV [106]. The codon alignments were examined for clock-like behaviour using the MrBayes strict-clock model. Speciation or duplication events were inferred using the method of Zmasek and Eddy [107].

To corroborate the Bayesian results [108], maximum likelihood analyses of the codon alignments and maximum parsimony and neighbour-joining analyses of the aa alignments with 1000 bootstrap replicates were conducted using PAUP 4.0b10 [109]. In order to understand the evolution of the sub-domain structure of Vtgs, the ratio of non-synonymous (Ka) to synonymous (Ks) nucleotide substitution rates were estimated using the Ka/Ks web service at the computational biology unit, University of Bergen [110], [111].

### Vitellogenin Gene Nomenclature

**Figure 1:**
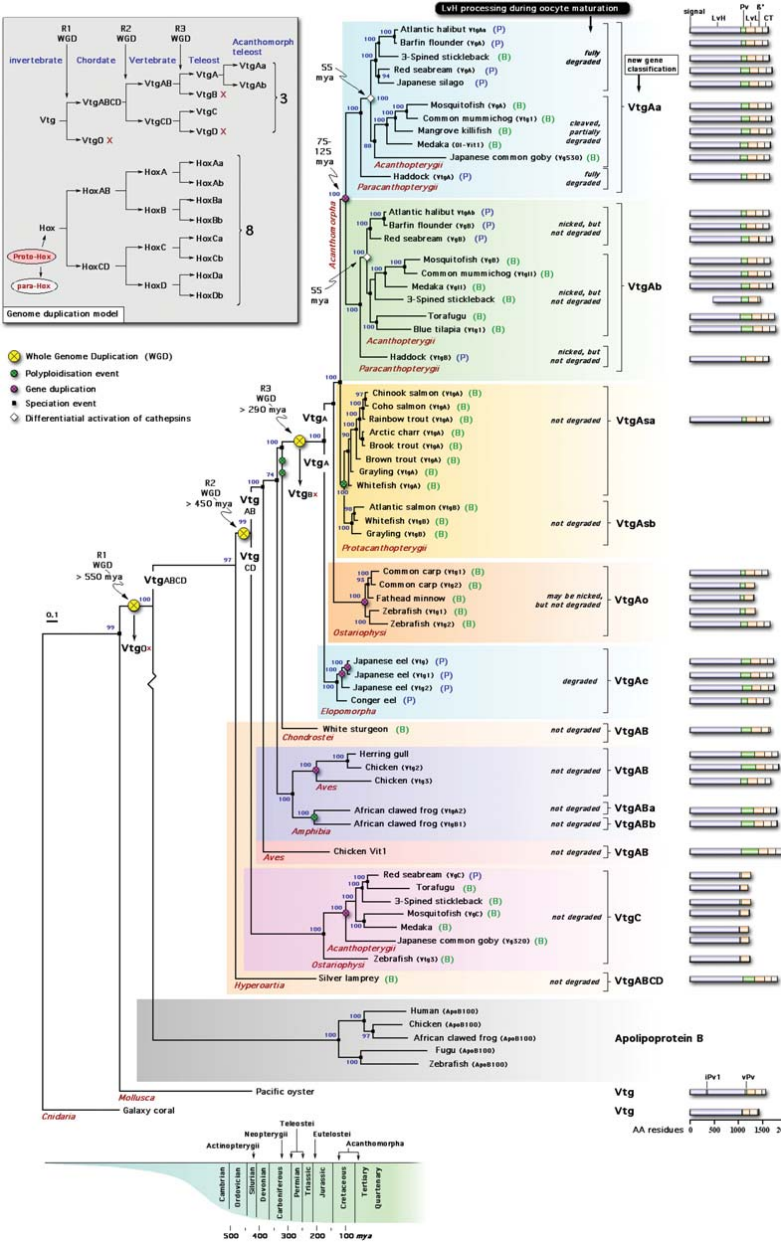
Bayesian majority rule consensus phylogenetic tree for the amino acid alignment of vertebrate large lipid transfer modules rooted with galaxy coral. Numbers beside nodes indicate posterior probabilities (consensus of 6,500 trees from 650,000 generations). See methods for further details of phylogentic analyses and supplementary material (Table S1) for accession numbers. Schematic linear scale representations of the structure of the vitellogenin genes are drawn for each taxa with complete sequences in the databanks. Sub-domain structures were identified from conserved cleavage sites. The iPv1 of Pacific oyster is proposed to be the origin of the first polyserine domain in the vitellogenins of insects. The geological scale provides an estimate of the divergence of the genes. Since the Vtg genes were not clock-like, this scale is approximate. See text and Fig. 2 for an explanation of the gene duplication model. X = gene lost; (P) = pelagic egg; (B) = benthic egg.

**Figure 2 pone-0000169-g002:**
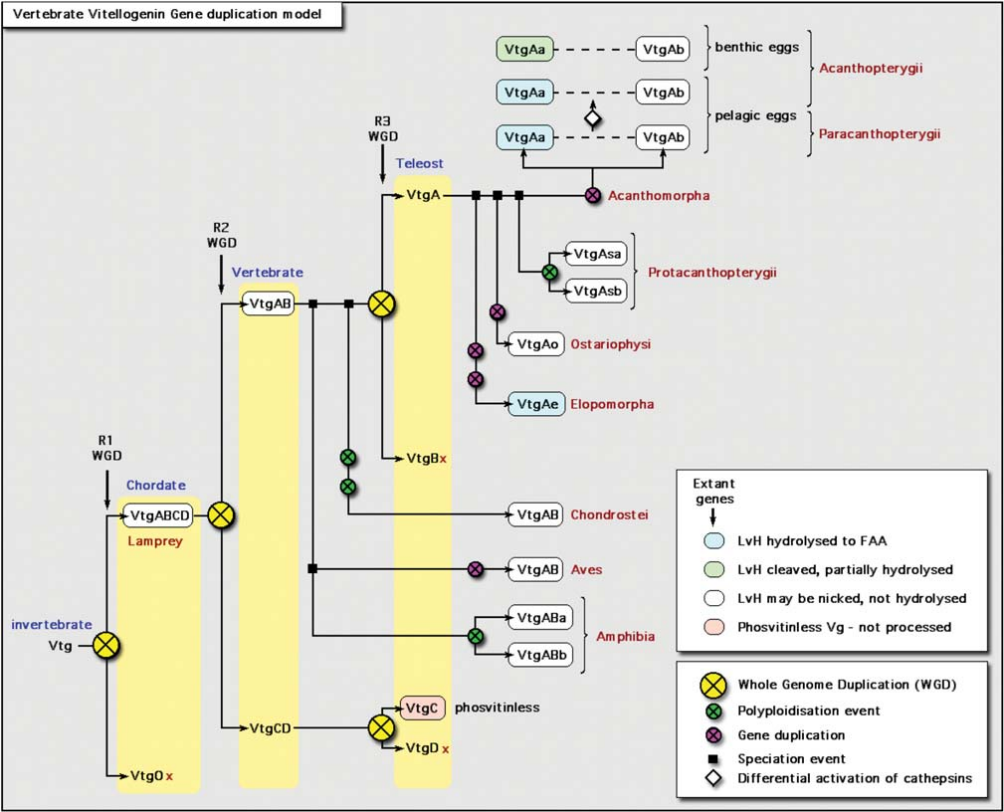
Proposed scheme for the classification of vertebrate vitellogenin genes based on three rounds (R1, R2, R3) of whole genome duplications (WGD), and lineage-specific gene duplications. The genes are classified according to their cluster pattern in Fig. 1 and Fig. 3 and the differential degradation of the lipovitellin heavy chain (LvH) during oocyte maturation. The loss of VtgD as is currently indicated in the scheme remains a logical conclusion since there is currently no evidence for its existence in the sequence or genome databases. Due to the low number of species examined to date, however, we do not preclude its existence. The white diamond within the acanthomorph lineage indicates differential regulation of cathepsins among pelagophils and benthophils.

In previous reports cloned teleost cDNAs encoding Vtgs with >1600 aa have been considered complete and classified as either (Vg/Vit), Vtg1, VtgI or VtgA and Vtg2, VtgII or VtgB, while cloned teleost cDNAs encoding Vtgs with <1400 aa and lacking a polyserine domain have been considered incomplete and classified as either Vtg3, VtgIII or (Vg)VtgC [65], [67], [72], [74], [93], [95], [112]–[116]. This nomenclature sometimes mixed between the types of Vtg, and sometimes placed the Vtgs of more basal teleosts with higher teleosts without establishing whether the genes were orthologous. Based on the Bayesian phylogenies that were supported by 100% posterior probability at 82% of the nodes and by >95% posterior probability at 95% of the nodes, we reclassified the gene nomenclature according to WGD, lineage-specific gene duplications and the functional properties of the LvH domains (see Fig. 1, Fig. 2 and supplementary information, Table S1).

## Results and Discussion

### Evolution of Vertebrate Vitellogenin Genes

As in previous studies [90], [91], [117] we found good homology between the LLTMs of vertebrate LvHs and apoBs, and for the separate vWFD domains (see supplementary material, Fig. S2 for alignment). Computed Ka/Ks ratios for each of these domains over the whole tree were not significantly different (0.411±0.181 and 0.375±0.177 between the LvH and vWFD domains, respectively), but were significantly lower (p<0.05) than for the LvL domains (0.492±0.163). Excluding the Pv domain, these Ka/Ks ratios indicate that the LvL domain is the least conserved, and that vertebrate Vtgs are not under strong purifying selection [110], [118], [119], but are evolving. This notion is corroborated by the Bayesian strict-clock model analyses, which showed that the evolution of the Vtg genes is not clock-like.

Although apoB is known to be related to Vtgs [89]–[91], the N-terminal LLTM has yet to be examined in the context of WGD. To investigate whether apoB could have arisen as a consequence of R1, we accessed the NCBI and Ensembl databases for related vertebrate and invertebrate lipoproteins. 128 of these sequences were used in an alignment consisting of ∼100 000 aa for Bayesian analysis. We followed the methodology as described above. These studies revealed that apoB is not a directly duplicated product of Vtg, but is secondarily derived from microsomal triglyceride transfer protein. To illustrate this, we included a kink in the descending branch of the tree that leads to the apoB cluster (Fig. 1).

Consensus Bayesian reconstruction of the evolution of vertebrate Vtg proteins (Fig. 1) was well supported by codon analyses (data not shown), and was congruent with both the fossil record [35]–[37] and current theory of vertebrate phylogeny [38], [39], [42]–[44], [46], [47], [120]. The great majority of nodes were supported by 100% posterior probability providing a solid statistical basis for interpretation of the tree topology. Moreover, switching between the cnidarian and molluscan outgroups did not alter the topology of the tree, and firmly established silver lamprey at the root of the vertebrate Vtgs. This finding reveals that the phosvitinless class of Vtgs, which to date has only been reported in teleosts, is more closely related to the Vtgs of vertebrates rather than insects. We propose that the phosvitinless class of Vtg is a neo-functional product of R2 (Fig. 2, and see later). The other methods of phylogenetic inference, maximum parsimony of the proteins, and maximum likelihood of the codons, also corroborated these findings (data not shown).

To reconcile the multiplicity of extant Vtg genes with the “3R hypothesis” of WGD we used a discontinuous evolutionary model of the Hox gene clusters in accordance with the literature [5]–[7], [31], [121]–[126]. Apparently, an ancient proto-Hox gene diverged to a para-Hox system and the Hox system [121], [123], [125], and the latter underwent several cis-duplications (intrachromosomal) yielding up to 13–14 genes before undergoing trans-duplications (interchromosomal) during R1, R2 and R3 to give rise to two, four and eight Hox clusters, respectively. Subsequent loss of genes has confused this pattern. For example until very recently zebrafish was thought to have lost the HoxDb cluster [124], but this has now been found in a degenerate form [127]. The HoxDb cluster is present in the medaka and torafugu, but these species have each apparently lost the HoxCb cluster [6]. Thus eight Hox clusters are known for the teleosts, while four are known in tetrapods, and only one Hox cluster is known in the early chordate ancestors (cephalochordates and urochordates). The presence of two Hox clusters for lamprey is congruent with scenario B proposed by Irvine *et al*. [128], and the observation that even though up to four clusters may exist in the Hyperoartia, they are not orthologous to the gnathostomes and hence duplicated independently [129]. We have therefore adopted an (AB)(CD) system for effects of WGD on the Vtg gene in accordance with Hox gene nomenclature. We recognize that the results of autotetrapoloidy followed by variable rates of diploidization may not generate symmetrical trees [17], and that Hox gene nomenclature was established through the chronology of discovery, rather than by phylogenetic analyses as is presently proposed for the Vtg genes. We further recognize that the ancestral vertebrate containing two Hox-clusters remains a logical hypothesis [6], [122].

Since the Vtg genes have the same longevity and metazoan heritage as the Hox clusters, there should be four Vtg genes in tetrapods, and eight Vtg genes present in teleosts. To date, up to four Vtg genes are known in amphibians (African clawed frog), of which two are sequenced [130], [131] and included in the tree. Currently three Vtg gene fragments are annotated in the genome of Western clawed frog (www.ensembl.org). The multiplicity of the Vtg system in amphibians, however, is the consequence of their tetraploidy [92] rather than the heritage of a WGD. In birds, three Vtg genes are known for the chicken, but all three are linked on chromosome 8 (www.ensembl.org). The major and minor Vtg genes, VtgAB2 and VtgAB3, respectively (see supplementary material Table S1), are known to have cis-duplicated and formed pseudogenes [132], and are co-located ∼2 Mbp upstream of VtgAB1. Interestingly, VtgAB1 does not cluster with the other avian Vtg genes, indicating that it has diverged functionally.

In teleosts, only three forms of Vtg have been found, which indicates that possibly up to five have been lost. Using the heuristic maxim of Occam's razor, we believe a more parsimonious explanation would be loss of a single Vtg gene paralogue (Vtg0) after R1 (see Fig. 1). This suggestion is semi-compatible with the arguments of von Shantz *et al*. [20], who, based on paralogon analyses of the circadian clock *Period* genes, concluded that one paralogue (*Per4*) was lost during the early rounds of WGD. Since there is greater similarity between *Per1* and *Per2* compared to *Per3* [133], the loss of *Per4* may have occurred after R2 rather than R1. This remains open to interpretation, however, since von Shantz *et al*. [20] concluded that the data did not conclusively support one pairing above others.

**Figure 3:**
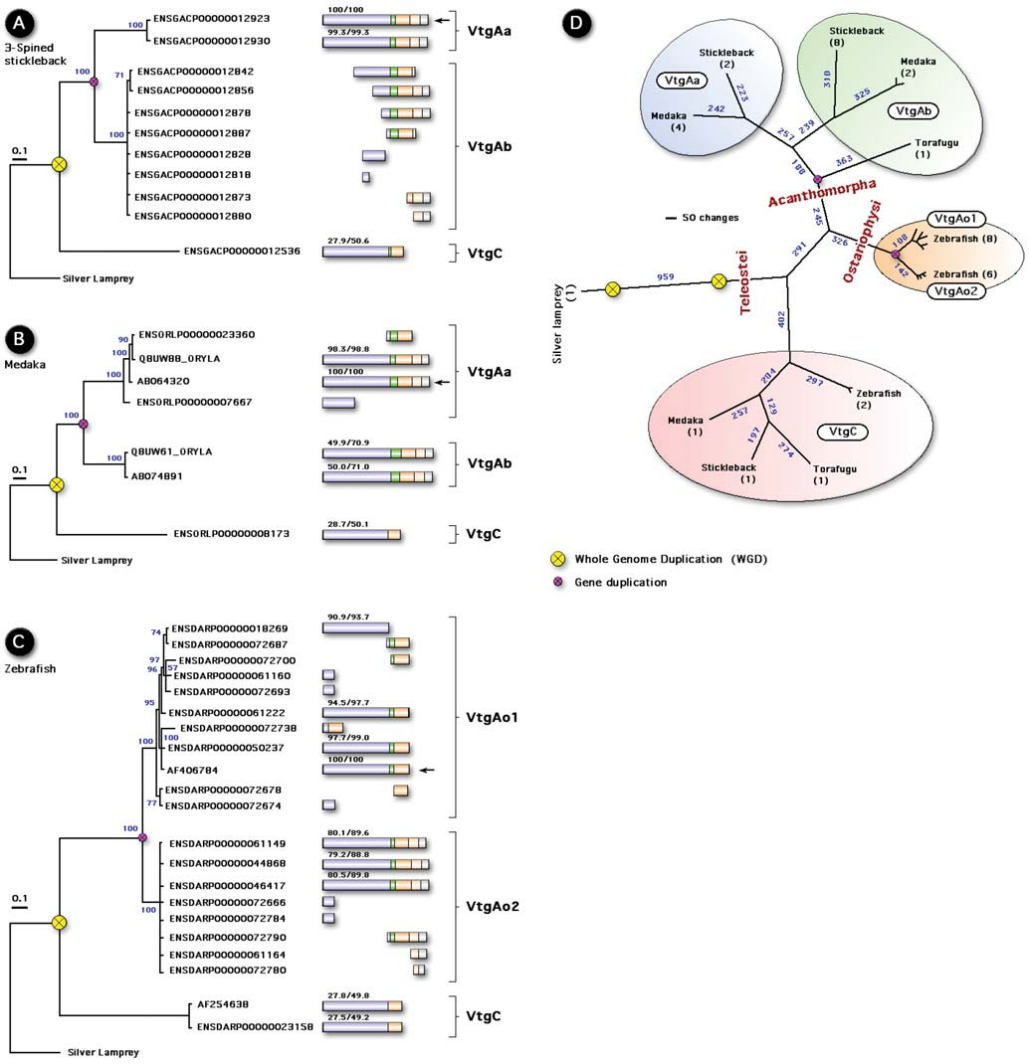
Bayesian majority rule consensus phylogenetic trees for the amino acid alignment of known and novel genomic vitellogenins. Panel A: 3-spined stickleback (*Gasterosteus aculeatus*); Panel B: Medaka (*Oryzias latipes*); Panel C: Zebrafish (*Danio rerio*) includes sequenced genes, and full genes ensembl release 38 and fragments from ensembl release 41; Panel D: Maximum parsimony phylogram of all known teleost genomic vitellogenin genes rooted with silver lamprey. The Vtg bars were constructed from conserved cleavage sites as described in Fig. 1. Homology identity and similarities for the full LvH domains are shown above each bar based on comparison to the reference sequence (arrow). See text and methods for further details of phylogentic analyses.

In the present context, the proposed loss of Vtg0 would explain the single gene in silver lamprey and the cluster of phosvitinless genes as the neo-functional product of R2. In support of this latter argument, it is noteworthy that phosvitinless Vtgs have not only lost the Pv domain, but also the ß ´ and CT domains comprising the vWFD (see Vtg bar structures, Fig. 1, Fig. 3). This is true for all currently sequenced VtgC forms, and those present in available teleost genomes. The propensity to lose domains is not restricted to the phosvitinless class of Vtg. Current evidence for genomic Vtg genes (3-spined stickleback, medaka and zebrafish) indicates that several fragments or truncated genes are present (see Fig. 3). Among the Ostariophysi, the major Vtg genes of zebrafish, common carp, and fathead minnow (VtgAo1) have also lost the vWFD domain. In zebrafish up to seven genes, including one pseudogene have been reported [93], [98], however, many more Vtg genes appear to exist in the genome (www.ensembl.org). Fourteen of these genes are tightly linked on chromosome 22, while the phosvitinless gene is located on chromosome 11. The last gene is not yet localized. We initially subjected predicted full genes (ensembl release 38, April 2006) to Bayesian analysis to find two closely related clusters, VtgAo1 and VtgAo2 and a third branch containing the more distantly related phosvitinless gene. With release 41 (October 2006) many of the earlier predicted genes are only present as fragments. We analyzed these fragments and compared them to the previously predicted genes to find that the three clusters VgAo1 (truncated), VgAo2 (complete), and VgC (phosvitinless) was supported by 100% posterior probabilities (Fig. 3c). Interestingly, the mRNA of the major gene, VtgAo1, is expressed 100–1000× higher than those of either the complete VtgAo2 or the phosvitinless form [98], suggesting that deleterious mutations have occurred in the cis-regulatory elements of the VtgAo2 genes. These analyses illuminate three points. The first is that Vtgs other than the phosvitinless class can lose domains. The second is that Vtg gene forms may be present in the genome, but are not significantly expressed, while the third strengthens the probability that the phosvitinless class is indeed a product of R2 since it is located on a separate chromosome. A caveat to the third point is that the VtgC genes of 3-spined stickleback and medaka are currently located on the same group (stickleback: group VIII) or chromosome (medaka: chromosome 4) ∼0.5–0.7 Mb from their VtgAa and VtgAb forms. However, since the chromosomal loci of the zebrafish VgC genes have been very recently reallocated from chromosome 15 to chromosome 11 in the latest Ensembl release (41), and the genomes of 3-spined stickleback and medaka are newly released, further analyses will be necessary to establish the definitive gene maps. Despite this caveat, maximum parsimony analysis of all genomic Vtg genes supports the topology of the tree in Fig. 1, and the division of the major forms of Vtgs in teleosts (Fig. 3D).

Placing the R3 at the base of the crown group of teleosts as recently proposed by Crow *et al*. [31] illustrates why the timing of this WGD, or whether it in fact occurred, has remained controversial [9], [12], [15], [18], [22]–[25]. The gene products of a WGD should show paralogous gene clustering. However, below the level of the Protacanthopterygii (salmonids), the Vtg genes do not show paralogous clustering. One interpretation for this could be the differential retention of paralogues among the different groups yielding an asymmetric tree in which the Elopomorpha, Ostariophysi and Protacanthopterygii have retained one paralogue (VtgB), and the Acanthomorpha both paralogues (VtgA and VtgB). However, with the exception of the top cluster encompassing Atlantic halibut to Japanese silago (Fig. 1), the identities and similarities for the LvH of each of these groups (50–65%, and 70–80%, respectively; see supplementary information Fig. S3) suggest otherwise. Further, since no phylogenetic method yielded orthologous relationships between the Vtgs of either the Elopomorpha, Ostariophysi, Protacanthopterygii and the Acanthomorpha or paralogous clustering of the Vtg genes immediately following R3, we suggest VtgB has been lost, and that all post-R3 teleost Vtg genes are derived from an ancestral VtgA type.

Both the Elopomorpha and Ostariophysi have duplicated their Vtg genes, but our analyses show that this has occurred in a lineage-specific manner after the WGD (Fig. 1, Fig. 3C&D). Similarly, although the Vtg genes of the salmonids do show paralogous clustering, this is argued to be the consequence of an independent polyploidy event that occurred between 25–50 mya [134]. Our current data match the first tree for salmonid Vtgs proposed by Buisine *et al*. [113]. Interestingly, with the exception of catfish, which are also tetraploid, the eggs of salmonids are among the largest shed by any broadcast spawning teleost. The loss of a paralogue in the *Oncorhynchus* genera after separation from *Salmo* genera ∼25 mya [39] resulted in local duplications of up to 31 copies of the other paralogue [113], suggesting a possible compensation for the synthesis of the large amounts of yolk. Notably, the WGD at the base of the crown group of teleosts coincides with appearance of telolecithal eggs and meroblastic cleavage. With the exception of Coelacanthimorpha, all other osteichthyan fishes below the level of teleosts, have mesolecithal eggs and holoblastic cleavage.

Above the level of the Protacanthopterygii, all Vtg genes show paralogous clustering giving rise to the proposed VtgAa and VtgAb paralogues among the Paracanthopterygii and Acanthopterygii (Fig. 1). This matches the appearance of the Acanthomorpha in the fossil record 75–100 mya [35], [37] and their estimated origin 125 mya [39]. Our analyses also indicate that a second putative lineage-specific duplication of the VtgA genes could have occurred with the appearance of the Acanthopterygii 55 mya. This suggests that four genes should exist in these species, but to date only dual Vtgs are known. Since we cannot preclude the existence of silent, or near silent genes as found for zebrafish (VtgAo2), this latter event may somehow represent a linked duplication or reflects a diversification of the Aa and Ab paralogues. No data are currently available for the chromosomal loci of these genes in the Acanthomorpha, and further studies will be necessary to clarify this issue. Presently we have indicated that this clustering is the result of differential cathepsin activation, since benthophils also express the VtgAa gene, but either do not, or only partially degrade the LvH during oocyte maturation [73], [73,81].

Remarkably, the gene clustering was highly correlated to the pelagic or benthic character of the egg. Since most marine teleosts spawn pelagic eggs [85], and only three Vtg forms from marine pelagophils have been fully sequenced to date, we suggest that the present tree does not reflect the bias toward VtgAa type genes. The observation that only the VtgAa forms are predominantly degraded during oocyte maturation, implies that this form of the gene has evolved novel sensitivity to the developmentally regulated activation of acid hydrolases (see later).

The proposed scheme of WGD and lineage-specific duplication of the Vtg genes precisely matches the observed number of vertebrate Vtg genes in accordance with the discontinuous Hox model (Fig. 1 inset). For lineage-specific VtgA duplications that resulted in paralogues, we have adopted the a, b convention. To avoid confusion with the independent tetraploidization amongst the Protacanthopterygii, we used VtgAsa and VtgAsb to denote paralogous genes that were previously classified as Vtg-A and Vtg-B, respectively [113]. The homologous Vtg genes of the Elopomorpha and Ostariophysi are preliminary classified as VtgAe and VtgAo, respectively. Further sequencing will be necessary to better establish their homology associations.

### Neo-Functionalization of the Vtg Genes

The mechanism of hydration differs between benthophils and pelagophils. In benthophils, the degree of oocyte hydration is less dramatic [69], [135] [73] and mostly dominated by an increase in inorganic ions and the presence of taurine [62], [64], [79], while in pelagophils, oocyte hydration is driven by an increase in the FAA content followed by inorganic ions [56], [57], [61], [67], [69], [84], [136]. Recent studies of the structure and disassembly of the yolk proteins during oocyte hydration in Atlantic halibut (Finn, unpublished data) and other marine pelagophils have demonstrated that the origin of the FAA pool stems mostly from the LvH of the VtgAa paralogue [65], [67], [74]. As in the more ancestral VtgABCD of lamprey [103] and VtgAo1 of zebrafish [82], the VtgAb LvH may be nicked, but is essentially not degraded.

Following fertilization and formation of the yolk syncytial layer, the surviving VtgAa and VtgAb gene products (yolk proteins) are also degraded by cathepsins as substrate for the developing embryo during yolk resorption [137]–[139], while the FAA generated from the maturational degradation of VtgAa become the dominant catabolic substrate for energy metabolism during embryogenesis (reviewed by [68]). In this respect the VtgAb paralogues maintain their original yolk function, but the present and previously published data demonstrate a post-duplication neo-functionalization of the VtgAa paralogues. This notion is supported by Ka/Ks ratios of 0.415 verses 0.294 for the branches that lead to the VtgAa and VtgAb clusters, respectively. Intriguingly, the VtgAa paralogue expressed in benthophils may also be partially cleaved and hydrolyzed during oocyte maturation, while the VtgAb paralogue remains intact for the developing embryo [73]. Confirmation that the VtgAa genes have undergone neo-functionalization arises from observations that all of the yolk proteins, including those that are derived from the VtgAb clusters become exposed to the acid hydrolases during the hydration event. However, it is primarily the LvH of the VtgAa paralogues that is degraded.

Many investigations have observed the maturational disappearance of high molecular weight proteins or the appearance of the FAA pool in other species that spawn pelagic eggs, including the Elopomorpha [140]–[142]. Indeed the FAA content of a newly fertilized teleost egg may be regarded as the signature of the pelagic egg (reviewed by [68]). The observation that Elopomorpha also have pelagic eggs, that arise with the disappearance of a high molecular weight Lv (110 kD) and a concomitant increase in FAA [140]–[142] indicates that the same mechanism has evolved in this group. Up to three forms of Vtg are known in Elopomorpha (Fig. 1 and supplementary material, Table S1), but these genes are not orthologous to the Vtgs of Acanthomorpha or indeed any other teleost group. For the Japanese eel, the LvH of VtgAe1 and VtgAe2 are 98% identical, while the LvH of VtgAe3 is only 86% identical indicating that at least two forms of Vtg exist in this group. Similar to the dual Vtgs of the Acanthomorpha, only the 110 kD Lv disappears during oocyte maturation of the Japanese eel [140]. However, due to the non-orthologous nature of eel Vtgs, we suggest that the pelagic eggs in this group have arisen by convergent evolution.

### Solving the Water Problem

We argue that the solution realized by marine teleosts that spawn pelagic eggs is the generation of a large pool of organic osmolytes (FAA) that drive hydration of the oocyte while still protected within the maternal ovary. Interestingly, this adaptation is analogous to the oviparous Chondrichthyes that store the organic end-products of protein metabolism (urea and trimethylamine oxides) in their eggs [143]–[145]. The functional significance of these mechanisms conforms to the compatible osmolyte hypothesis [146]. Unlike charged ions, neutral amino acids, which dominate the FAA pool [57], [67], [69], [136], [141], [142], [147]–[151] do not compromise enzyme function. We suggest that in teleosts, the retention of the organic end products of protein degradation was made possible by a post-R3 lineage-specific duplication of their Vtg genes, and the differential activation of acid hydrolases during oocyte maturation. Instead of converting their depolymerized yolk protein products to urea and trimethylamine oxides, they retain them in a free form as FAA, which, due to their transiently increased concentration in the yolk relative to the maternal body fluids, drive hydration of the oocyte. Once the eggs are broadcast into the sea, the formation of the virtually impermeable vitelline membrane during the cortical reaction [152]–[156] prevents the loss of this water of life until osmoregulatory mechanisms develop during embryogenesis. The greater degradation of the VtgAa forms in pelagophils led to the highly hydrated egg and caused them to float, while the FAA comprise the major substrate that fuels embryonic development. This mechanism appears to have independently evolved in the Elopomorpha.

### Oceanic Radiation and Speciation

The greater hydration associated with the spawning of pelagic eggs (>90% water) in vast numbers would have severely loaded the maternal ovary with water, and probably led to batch spawning, which is the prevalent means of reproduction in the extant Acanthomorpha. We thus argue that the pelagic nature of the egg, which arose due to neo-functionalization of the Vtg paralogues, provided the allopatric means of radiation in the oceans, while the retention of 3R gene products, latently yielded the genetic means for adjustments in pattern formation and speciation. The rapid acanthomorph colonization of the oceans occurred when competition and predation was low [157], [158] following the Cretaceous-Tertiary boundary extinction. Such a lack of competition and reduced predation pressure may have provided the opportunity for a flotilla of teleost invaders in the oceans.

## Supporting Information

Figure S1 Phylogenetic organisation of the fishes illustrating the fraction of species spawning benthic (B) or pelagic (P) eggs, or having viviparous/ovoviviparous (V) reproduction in seawater or freshwater. A plus indicates that the mode of reproduction occurs in the given order. Estimates of minimum paleolontological dates or calculated divergence times (millions of years ago; mya) according to the fossil record or mitogenomic data (Inoue et al., 2005) are given for the appearance of the major groups. Model species that are currently undergoing complete genome sequencing belong to orders highlighted in grey.
(0.10 MB PDF)

Figure S2 Multiple sequence alignment of vertebrate vitellogenins and apolipoprotein B100. Reclassification of the genes as shown in Figs 1–3 in manuscript are indicated. Sub-domains of the vitellogenin monomers are indicated by labeled bars beneath the alignment. Conserved cleavage sites are annotated above the sequences. Data sets for phylogenetic analyses are illustrated by grey bars under the relevant domains.
(8.47 MB JPG)

Figure S3 Similarity and identity scores for the lipovitellin heavy chains in the multiple sequence alignment shown in Fig. S2. Cells are colored according to score.
(0.04 MB PDF)

Table S1 Accession numbers of sequences and taxa used in the analyses.
(0.06 MB PDF)
