# Supplementary material for: Vertebrate Vitellogenin Gene Duplication in Relation to the “3R Hypothesis”: Correlation to the Pelagic Egg and the Oceanic Radiation of Teleosts
Source: PLoS One. 2007 Jan 24;2(1):e169. doi: 10.1371/journal.pone.0000169 (PMC1770952; doi:10.1371/journal.pone.0000169)

Figure S1: Finn and Kristoffersen

Phylogenetic organisation of the fishes illustrating the fraction of species spawning benthic (B) or pelagic (P) eggs, or having viviparous/ovoviviparous (V) reproduction in seawater or freshwater. A plus indicates that the mode of reproduction occurs in the given order. Estimates of minimum paleolontological dates or calculated divergence times (millions of years ago; mya) according to the fossil record or mitogenomic data are given for the appearance of the major groups. Model species that are currently undergoing complete genome sequencing belong to orders highlighted in grey.

Sources

Inoue JG, Miya M, Tsukamoto K, Nishida M (2004) Mitogenomic evidence for the monophyly of elopomorph fishes (Teleostei) and the evolutionary origin of the leptocephalus larva. Mol Phylogen Evol 32: 274-

Inoue JG, Miya M, Venkatesh B, Nishida M (2005) The mitocondrial genome of Indoseian coelacanth Latimeria menadoensis (Sarcopterygii: Colacanthiformes) and divergence time estimation between the two coelacanths. Gene 349: 227-235

Kumazawa Y, Nishida M (2000) Molecular phylogeny of Osteoglossoids: A new model for Gondwanian origin and plate tectonic transportation of the asian aruwana. Mol Biol Evol 17: 1869-1878

Long JA (1995) The rise of fishes. 500 million years of evolution. The John Hopkins University Press, Baltimore

Maissey JG (1996) Discovering fossil fishes. Westview Press, New York

Moser HG (1983) Ontogeny and systematics of fishes. American Society Ichthyologists Herpetolologists Special publication Number 1. Symposium 15-18 Aug, La Jolla, California

Nelson JS (2006) Fishes of the world. John Wiley & Sons Inc, New York

Russell FS (1976) The eggs and planktonic stages of British marine fishes. Academic Press, New York

Stiassny MLJ, Parenti LR, Johnson GD (1996) Interrelationships of fishes. Academic Press, New York

Superclass	Class	Subclass	Infraclass	Subdivision	Order	B	P	V
>500 mya	Myxini				Myxiniformes	+		
Agnatha	Cephalaspidomorphi				Petromyzontiformes	+		
Gnathostomata >450 mya	Chondrichthyes >450 mya	Holocephali			Chimaeriformes	+		
		Elasmobranchii	Galeomorphii	Euselachii	Heterodontiformes	+		
					Orectolobiformes	+		+
					Carcharhiniformes	+		+
					Lamniformes			+
					Hexanchiformes			+
					Echinorhiniformes			+
			Squalea		Squaliformes			+
					Squatiniformes			+
					Pristiophoriformes			+
					Torpediniformes			+
					Pristiformes			+
					Rajiformes	+		
					Myliobatiformes			+
	Osteichthyes >450 mya	Crossopterygii		Coelacanthimorpha	Coelacanthiformes			+
		Dipneusti	Dipnoi	Ceratodontimorpha	Ceratodontiformes	+		
					Lepidosireniformes	+		
		Actinopterygii >400 mya	Chondrostei		Polypteriformes	+		
					Acipenseriformes	+		
			Neopterygii		Lepisosteiformes	+		
					Amiiformes	+		
			Teleostei >290 mya	Osteoglossomorpha	Hiodontiformes	+		
					Osteoglossiformes	+		
				Elopocephala: 230-260 mya Elopomorpha	Elopiformes		+	
					Albuliformes		+	
					Anguilliformes		+	
					Saccopharyngiformes	unknown		
				200-240 mya Clupeomorpha	Clupeiformes	+	+	
				Clupeocephala: 130-160 mya Ostariophysi	Gonorhynchiformes	+	+	
					Cypriniformes	+		
					Characiformes	+		
					Siluriformes	+		
					Gymnotiformes	+		
				Euteleostei: 80-110 mya Protacanthopterygii	Argentiniformes	+		
					Esociformes	+	+	
					Osmeriformes	+	+	
					Salmoniformes	+		
				Stenopterygii	Stomiiformes		+	
					Ateleopodiformes	unknown		
				Cyclosquamata	Aulopiformes		+	
				Scopelomorpha	Myctophiformes		+	
				Lampridimorpha	Lampriformes		+	
				Polymixiomorpha	Polymixiiformes	unknown		
				Acanthomorpha >75 mya: Paracanthopterygii	Percopsiformes	+		
					Ophidiiformes	+	+	+
					Gadiformes	+	+	
					Batrachoidiformes	+		
					Lophiiformes		+	
				Acanthomorpha: 55 mya Acanthopterygii	Mugiliformes	+	+	
					Atheriniformes	+		+
					Beloniformes	+	+	
					Cyprinodontiformes	+		+
					Stephanoberyciformes	unknown		
					Beryciformes	unknown		
					Zeiformes		+	
					Gasterosteiformes	+	+	
					Synbranchiformes	+		
					Scorpaeniformes	+	+	+
					Perciformes	+	+	
					Pleuronectiformes	+	+	
					Tetraodontiformes	+	+	

The percent distribution of fishes from 63 orders showing the relative sub-fraction spawning in seawater or freshwater

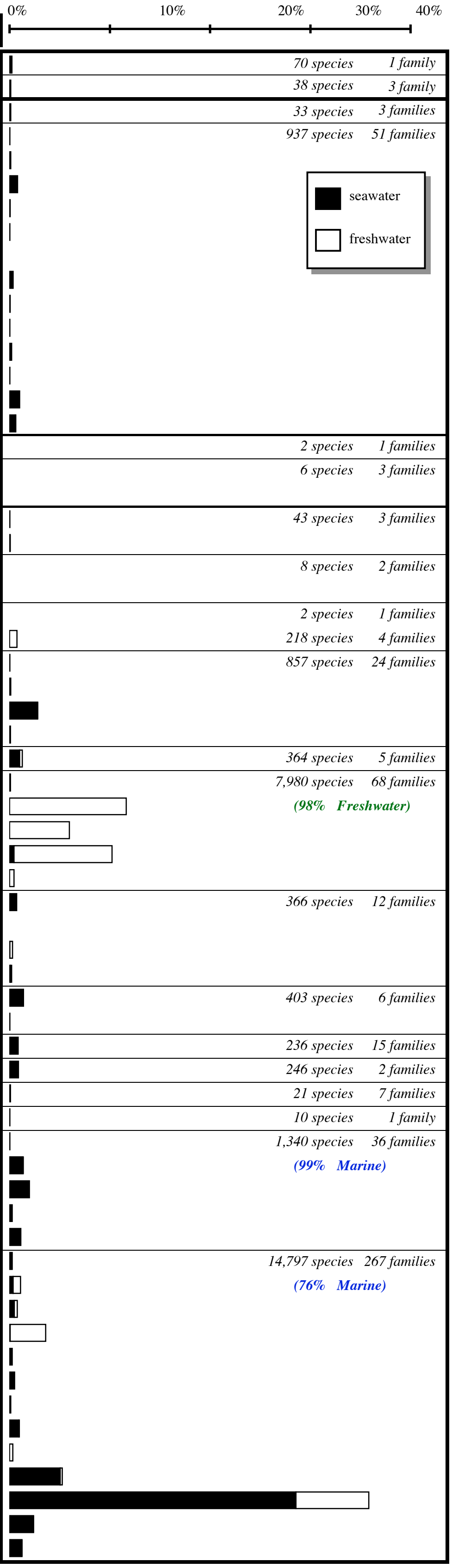

Supplement: Figure S1 — Phylogenetic organisation of the fishes illustrating the fraction of species spawning benthic (B) or pelagic (P) eggs, or having viviparous/ovoviviparous (V) reproduction in seawater or freshwater. A plus indicates that the mode of reproduction occurs in the given order. Estimates of minimum paleolontological dates or calculated divergence times (millions of years ago; mya) according to the fossil record or mitogenomic data (Inoue et al., 2005) are given for the appearance of the major groups. Model species that are currently undergoing complete genome sequencing belong to orders highlighted in grey. (0.10 MB PDF) [file pone.0000169.s001.pdf]
