# Supplementary material for: Vertebrate Vitellogenin Gene Duplication in Relation to the “3R Hypothesis”: Correlation to the Pelagic Egg and the Oceanic Radiation of Teleosts
Source: PLoS One. 2007 Jan 24;2(1):e169. doi: 10.1371/journal.pone.0000169 (PMC1770952; doi:10.1371/journal.pone.0000169)
Supplement: Figure S3 — Similarity and identity scores for the lipovitellin heavy chains in the multiple sequence alignment shown in Fig. S2. Cells are colored according to score. (0.04 MB PDF) [file pone.0000169.s003.pdf]

Fig S3: Finn and Kristoffersen

Similarity and identity scores for complete teleost vitellogenin lipovitellin heavy chains. Cells are coloured according to score

Identity Scores (%) (above diagonal); Similarity Scores (%) (below diagonal)

	Atlantic halibut vtgAa	AB181833 Barfin flounder	ENSGACP00000012923 3-spined Stickleback	AB181838 Red seabream	AB081299 Japanes silago	AB181835 Mosquitofish	U07055 Common mummichog	AY279214 Mangrove killifish	AB064320 Medaka	AB088473 Japanese common goby	AF284035 Haddock	Atlantic halibut vtgAb	AB181834 Barfin flounder	AB181839 Red seabream	AB181836 Mosquitofish	FHU70826 Common mummichog	AB074891 Medaka	NEWSINFRUP00000179080 Torafugu	AF017250 Blue tilapia	AF284034 Haddock	X92804 Rainbow trout	AB106873 Common carp	AF414432 Common carp	AF130354 Fathead minnow	AF406784 Zebrafish	ENSDARP00000044868 Zebrafish	AY775788 Japanese eel	AY423445 Japanese eel	AY423444 Japanese eel	AB185334 Conger eel
**Atlantic halibut vtgAa**		97	73	77	75	60	63	64	63	50	63	60	60	60	51	51	56	48	52	58	61	56	54	55	53	51	56	56	55	55
**AB181833 Barfin flounder**	99		73	78	75	60	63	64	63	50	63	60	60	60	51	52	55	48	52	58	61	56	54	55	53	51	56	57	55	56
**ENSGACP00000012923 3-spined Stickleback**	86	86		76	72	58	61	61	60	49	62	58	58	57	50	50	52	46	50	55	58	53	52	52	51	49	53	54	52	52
**AB181838 Red seabream**	90	90	86		80	62	65	66	65	51	67	60	59	60	52	53	55	48	52	58	63	57	56	57	55	52	57	58	57	58
**AB081299 Japanes silago**	88	88	86	91		61	63	63	63	50	65	60	60	60	51	53	55	47	51	58	61	57	55	56	55	53	57	57	56	57
**AB181835 Mosquitofish**	77	77	75	78	79		77	68	62	43	55	52	52	52	47	46	49	43	45	50	53	51	50	50	49	47	51	52	50	51
**U07055 Common mummichog**	78	78	76	79	79	87		73	66	45	58	55	55	54	49	48	50	46	46	53	55	52	51	52	51	48	51	51	50	50
**AY279214 Mangrove killifish**	78	78	76	80	79	81	84		66	45	58	53	54	53	47	46	49	46	47	52	54	51	50	51	48	48	51	52	50	51
**AB064320 Medaka**	78	77	76	79	79	78	79	79		46	58	52	52	52	47	47	49	44	45	51	55	51	50	52	50	48	50	50	49	50
**AB088473 Japanese common goby**	68	68	67	68	69	64	64	64	65		47	44	44	42	40	39	40	37	40	42	43	43	43	44	41	41	42	42	41	41
**AF284035 Haddock**	82	82	79	84	84	75	76	76	77	68		59	58	58	51	51	55	48	51	56	60	55	54	54	52	50	56	56	55	55
**Atlantic halibut vtgAb**	77	77	76	78	78	71	72	71	72	63	77		96	80	63	65	68	58	63	69	64	58	58	58	56	53	59	59	56	58
**AB181834 Barfin flounder**	77	77	76	78	78	71	72	71	72	64	76	99		80	63	64	67	57	62	68	62	57	57	57	55	52	58	58	56	58
**AB181839 Red seabream**	77	78	75	78	78	72	72	72	72	64	77	90	90		62	63	69	59	65	70	64	57	56	56	55	52	60	60	58	60
**AB181836 Mosquitofish**	71	72	71	73	72	68	69	67	68	61	71	81	81	81		80	65	51	56	58	54	51	51	52	51	48	52	52	50	52
**FHU70826 Common mummichog**	72	72	71	73	73	67	69	67	69	60	72	82	82	82	89		67	52	56	58	54	51	51	52	51	48	52	52	50	52
**AB074891 Medaka**	74	74	72	75	75	69	70	68	70	62	74	85	85	86	83	85		53	58	62	58	53	53	52	52	50	54	55	53	54
**NEWSINFRUP00000179080 Torafugu**	68	69	67	69	68	63	64	65	64	58	68	75	75	76	70	71	73		51	54	51	48	47	47	47	46	47	48	47	47
**AF017250 Blue tilapia**	71	72	71	72	73	67	68	67	67	60	72	81	81	83	77	78	79	72		59	55	51	51	51	50	48	52	52	50	52
**AF284034 Haddock**	76	76	74	77	76	70	72	71	71	62	74	85	85	86	78	78	81	73	79		61	55	54	54	53	51	57	57	56	58
**X92804 Rainbow trout**	77	77	75	78	78	71	71	71	72	63	78	79	79	80	74	74	78	69	74	79		62	60	60	60	56	64	64	62	63
**AB106873 Common carp**	75	75	74	76	76	70	71	70	71	63	74	77	77	75	72	72	73	68	71	74	77		89	86	82	76	59	60	58	59
**AF414432 Common carp**	74	74	73	76	76	70	71	70	71	63	74	77	76	75	71	72	73	67	71	74	76	94		90	84	77	59	59	57	59
**AF130354 Fathead minnow**	74	74	73	75	76	70	71	70	72	63	74	77	77	75	72	72	73	67	71	74	76	92	95		84	77	60	60	58	59
**AF406784 Zebrafish**	73	73	72	74	74	69	70	69	71	62	72	75	75	74	71	71	73	67	70	73	76	90	92	91		79	58	58	56	56
**ENSDARP00000044868 Zebrafish**	70	70	69	71	72	66	66	67	69	60	70	73	73	71	68	68	70	65	68	70	71	87	87	86	88		55	55	53	54
**AY775788 Japanese eel**	74	74	72	75	75	70	70	71	71	62	75	76	76	77	72	73	75	68	72	76	78	75	75	76	74	72		98	86	83
**AY423445 Japanese eel**	74	75	73	76	75	71	71	71	71	63	75	76	76	77	73	74	76	68	73	76	78	76	76	77	75	72	98		86	84
**AY423444 Japanese eel**	73	73	71	74	74	69	69	70	69	61	73	74	74	75	70	71	73	66	70	74	77	74	74	75	73	70	90	91		81
**AB185334 Conger eel**	74	74	72	76	75	70	70	71	71	62	75	76	76	77	73	73	75	67	72	76	78	76	76	76	75	72	90	90	89	

>50%   >70%   >85%
