# Supplementary material for: Vertebrate Vitellogenin Gene Duplication in Relation to the “3R Hypothesis”: Correlation to the Pelagic Egg and the Oceanic Radiation of Teleosts
Source: PLoS One. 2007 Jan 24;2(1):e169. doi: 10.1371/journal.pone.0000169 (PMC1770952; doi:10.1371/journal.pone.0000169)
Supplement: Table S1 — Accession numbers of sequences and taxa used in the analyses. (0.06 MB PDF) [file pone.0000169.s004.pdf]

Table S1: Supplementary material: Finn and Kristoffersen

Accession numbers of sequences and taxa used in the analyses

	Accession #	Database	Classified					Length		
	(NCBI / Ensembl)	ID	homologue	Rank/Class	Order	Animal	Species	bp	aa	Source
1	HUMAPOBB	apo-B100	apoB100	Eutheria	Primates	Human	*Homo sapiens*	13,689	4,563	Law et al. (1986)
2	ENSGALP00000026550	apo-B100	apoB100	Aves	Galliformes	Chicken	*Gallus gallus*	12,267	4,089	Ensembl (2006)
3	D89547	Vit1	vtgAB1			Chicken	*Gallus gallus*	5,736	1,912	Mabuchi et al. (1999)
4	NM_001031276	vtgII	vtgAB2			Chicken	*Gallus gallus*	5,550	1,850	van het Schip et al. (1999)
5	ENSGALT00000002890	novel	vtgAB3			Chicken	*Gallus gallus*	5,058	1,686	Ensembl (2006)
6	AY045719	vitellogenin	vtgAB		Charadriiformes	Herring gull	*Larus argentatus*	5,496	1,832	Lorenzen et al. (2001)
7	AAH54229	apo-B100	apoB100	Amphibia	Anura	African clawed frog	*Xenopus laevis*	1,311	437	Klein et al. (2002)
8	M18061	vtgA2	vtgABa			African clawed frog	*Xenopus laevis*	5,421	1,807	Gerber-Huber et al. (1987); Nardelli et al. (1987)
9	AB092605	vtgb1	vtgABb			African clawed frog	*Xenopus laevis*	5,451	1,817	Yoshitome et al. (2003)
10	NEWSINFRUP00000127842	apo-B100	apoB100	Acanthopterygii	Tetraodontiformes	Torafugu	*Takifugu rubripes*	11,145	3,715	Ensembl (2006)
11	NEWSINFRUP00000179080	novel	vtgAb			Torafugu	*Takifugu rubripes*	5,274	1,758	Ensembl (2006)
12	NEWSINFRUP00000131263	novel	vtgC			Torafugu	*Takifugu rubripes*	3,594	1,198	Ensembl (2006)
13	hhvtgAa1	vtgAa1	vtgAa		Pleuronectiformes	Atlantic halibut	*Hippoglossus hippoglossus*	4,899	1,633	Finn et al. (2006)
14	hhvtgAb1	vtgAb1	vtgAb			Atlantic halibut	*Hippoglossus hippoglossus*	4,941	1,647	Finn et al. (2006)
15	AB181833	VgA	vtgAa			Barfin flounder	*Verasper moseri*	4,884	1,628	Sawaguchi et al. (2005)
16	AB181834	VgB	vtgAb			Barfin flounder	*Verasper moseri*	4,953	1,651	Sawaguchi et al. (2005)
17	AF017250	Vtg1	vtgAb		Perciformes	Blue Tilapia	*Oreochromis aureus*	5,364	1,788	Lim et al. (1997)
18	AB088473	Vg-530	vtgAa			Japanese common goby	*Acanthogobius flavimanus*	4,992	1,664	Ohkubo et al. (2004)
19	AB088474	Vg-320	vtgC			Japanese common goby	*Acanthogobius flavimanus*	3,714	1,238	Ohkubo et al. (2004)
20	AB081299	VgA	vtgAa			Japanese silago	*Sillago japonica*	5,103	1,701	Yoon et al. (2002)
21	AB181838	VgA	vtgAa			Red seabream	*Pagrus major*	5,145	1,715	Sawaguchi et al. (2006)
22	AB181839	VgB	vtgAb			Red seabream	*Pagrus major*	5,148	1,716	Sawaguchi et al. (2006)
23	AB181840	VgC	vtgC			Red seabream	*Pagrus major*	3,816	1,272	Sawaguchi et al. (2006)
24	ENSGACP00000012923	novel	vtgAa		Gasterosteiformes	3-spined stickleback	*Gasterosteus aculeatus*	4,962	1,654	Ensembl (2006)
25	ENSGACP00000012842	novel	vtgAb			3-spined stickleback	*Gasterosteus aculeatus*	2,952	984	Ensembl (2006)
26	ENSGACP00000012536	novel	vtgC			3-spined stickleback	*Gasterosteus aculeatus*	3,813	1,271	Ensembl (2006)
27	AB181835	VgA	vtgAa		Atheriniformes	Mosquitofish	*Gambusia affinis*	5,085	1,695	Sawaguchi et al. (2005)
28	AB181836	VgB	vtgAb			Mosquitofish	*Gambusia affinis*	5,025	1,675	Sawaguchi et al. (2005)
29	AB181837	VgC	vtgC			Mosquitofish	*Gambusia affinis*	3,726	1,242	Sawaguchi et al. (2005)
30	AB064320	Ol-vit1	vtgAa		Beloniformes	Medaka	*Oryzias latipes*	5,034	1,678	Arai et al. (2001); Fujiwara et al. (2002)
31	AB074891	vgII	vtgAb			Medaka	*Oryzias latipes*	5,175	1,725	Murakami and Nakai (2003)
32	ENSORLP00000008173	novel	vtgC			Medaka	*Oryzias latipes*	3,663	1,221	Ensembl (2006)
33	AY279214	vtg	vtgAa		Cyprinodontiformes	Mangrove killifish	*Kryptolebias marmoratus*	5,133	1,711	Kim et al. (2004)
34	U07055	vtgI	vtgAa			Common mummichog	*Fundulus heteroclitus*	5,112	1,704	LaFleur et al. (1995)
35	FHU70826	vtgII	vtgAb			Common mummichog	*Fundulus heteroclitus*	5,061	1,687	LaFleur et al. (1995)
36	AF284035	VtgA	vtgAa	Paracanthopterygii	Gadiformes	Haddock	*Melanogrammus aeglefinus*	4,995	1,665	Reith et al. (2001)
37	AF284034	VtgB	vtgAb			Haddock	*Melanogrammus aeglefinus*	4,929	1,643	Reith et al. (2001)
38	AF454748	VtgA	vgAsa	Protacanthopterygii	Salmoniformes	Chinook salmon	*Oncorhynchus tshawytscha*	804	268	Buisine et al. (2002)
39	AF454747	VtgA	vgAsa			Coho Salmon	*Oncorhynchus kisutch*	807	269	Buisine et al. (2002)
40	X92804	vtg1	vgAsa			Rainbow trout	*Oncorhynchus mykiss*	4,977	1,659	Mouchel et al. (1996)
41	AF454751	VtgA	vgAsa			Artic charr	*Salvelinus alpinus*	810	270	Buisine et al. (2002)
42	AF454752	VtgA	vgAsa			Brook trout	*Salvelinus fontinalis*	807	269	Buisine et al. (2002)
43	AF454749	VtgB	vgAsb			Atlantic salmon	*Salmo salar*	849	283	Buisine et al. (2002)
44	AF454750	VtgA	vgAsa			Brown trout	*Salmo trutta*	807	269	Buisine et al. (2002)
45	AF454745	VtgA	vgAsa			Whitefish	*Coregonus lavaretus*	807	269	Buisine et al. (2002)
46	AF454746	VtgB	vgAsb			Whitefish	*Coregonus lavaretus*	807	269	Buisine et al. (2002)
47	AF454753	VtgA	vgAsa			Grayling	*Thymallus thymallus*	807	269	Buisine et al. (2002)
48	AF454754	VtgB	vgAsb			Grayling	*Thymallus thymallus*	807	269	Buisine et al. (2002)
49	AF414432	vitellogenin	vtgAo1	Ostariophysi	Cypriniformes	Common carp	*Cyprinus carpio*	4,059	1,353	Chang et al. (2001); Lai et al. (2001)
50	AB106873	vitellogenin	vtgAo2			Common carp	*Cyprinus carpio*	4,872	1,624	Han et al. (2004)
51	AF130354	vtg	vtgAo1			Fathead Minnow	*Pimephales promelas*	4,017	1,339	Korte et al. (2000)
52	ENSDARP00000062792	apo-B100	apoB100			Zebrafish	*Danio rerio*	11,346	3,782	Ensembl (2006)
53	AF406784	vtg1	vtgAo1			Zebrafish	*Danio rerio*	4,086	1,362	Islinger et al. (2003)
54	ENSDARP00000018269	novel	vtgAo1			Zebrafish	*Danio rerio*	3,147	1,049	Ensembl (2006)
55	ENSDARP00000061222	novel	vtgAo1			Zebrafish	*Danio rerio*	4,977	1,659	Ensembl (2006)
56	ENSDARP00000044868	novel	vtgAo2			Zebrafish	*Danio rerio*	5,010	1,670	Ensembl (2006)
57	ENSDARP00000046417	novel	vtgAo2			Zebrafish	*Danio rerio*	5,022	1,674	Ensembl (2006)
58	ENSDARP00000061149	novel	vtgAo2			Zebrafish	*Danio rerio*	4,890	1,630	Ensembl (2006)
59	ENSDARP00000061160	novel	vtgAo2			Zebrafish	*Danio rerio*	5,061	1,687	Ensembl (2006)
60	ENSDARP00000061164	novel	vtgAo2			Zebrafish	*Danio rerio*	5,064	1,688	Ensembl (2006)
61	AF254638	vg3	vtgC			Zebrafish	*Danio rerio*	3,753	1,251	Wang et al. (2000)
62	AY775788	Vtg	vtgAe1	Elopomorpha	Anguilliformes	Japanese eel	*Anguilla japonica*	5,226	1,742	Wang and Lou (2006)
63	AY423445	VTG1	vtgAe2			Japanese eel	*Anguilla japonica*	5,202	1,734	Okumura et al. (2003); Mikawa et al. (2006)
64	AY423444	VTG2	vtgAe3			Japanese eel	*Anguilla japonica*	5,277	1,759	Okumura et al. (2003); Mikawa et al. (2006)
65	AB185334	vtg	vtgAe1			Conger eel	*Conger myriaster*	4,989	1,663	Mikawa et al. (2006)
66	U00455	vitellogenin	vtgAB	Chondrostei	Acipenseriformes	White Sturgeon	*Acipenser transmontanus*	5,031	1,677	Bidwell and Carlson (1995)
67	M88749	vitellogenin	vtgABCD	Hyperoartia	Petromyzontiformes	Silver lamprey	*Ichthyomyzon unicuspus*	5,469	1,823	Sharrock et al. (1992)
68	AB084783	vg	vtg	Mollusca	Ostreoida	Pacific oyster	*Crassostrea gigas*	4,749	1,583	Matsumoto et al. (2002)
69	AB179781	Vg	vtg	Anthozoa	Scleractinia	Galaxy Coral	*Galaxea fascicularis*	4,317	1,439	Hayakawa et al. (2004; 2006)
